# Ideologies of Political Constitutionalism

**DOI:** 10.1093/ojls/gqae028

**Published:** 2024-09-04

**Authors:** Robert Greally

**Keywords:** public law, constitutional theory, political constitutionalism, political ideologies, democracy, UK constitution

## Abstract

For many political constitutionalists, the ordinary democratic process should be the constitution; constitutional entrenchment and strong-form judicial review should be avoided. But how is ordinary democratic politics understood by political constitutionalists? To answer this question, this article engages in an interpretative inquiry to delineate four distinct ideological readings of political constitutionalism—democratic socialist, liberal, republican and conservative—that are alive within the existing literature. It does so to explain how these readings articulate subtly different understandings of ordinary democratic politics. In doing so, it reflects on how to identify political constitutionalist thought; how political constitutionalism can appeal to different ideologies; how ideologies have influenced the theory’s intellectual development; and the ideological conditions required to sustain a political constitution.

## 1. Introduction

How is ordinary democratic politics understood by political constitutionalists? For many political constitutionalists, the ordinary democratic process is and should be the constitution. They advocate a distinctive vision of a constitutional order in which the exercise of state power is primarily organised around and regulated by ordinary democratic politics rather than an entrenched constitution guarded by the judiciary. Yet, political constitutionalist claims about how the ordinary democratic process should operate and the kinds of constitutional goods it should seek to promote are often vague. This article adopts a novel approach to answer this question by delineating within the existing literature between four distinct ideological readings of political constitutionalist thought—*democratic socialist*, *liberal*, *republican* and *conservative*—to explain how these readings articulate subtly different understandings of ordinary democratic politics. It posits that although the theory of political constitutionalism may aspire to be as prescriptively minimal as possible,^1^ the various concepts and processes associated with ordinary democratic politics ultimately acquire more specific and substantive meanings when understood through the prism of an adherent’s specific political ideology. By studying the influence of ideologies on political constitutionalist thought, we can see how different ideologies place different stresses upon aspects of democratic politics. This explains why political constitutionalists often respond differently to contemporary issues about the UK’s predominately political constitution. Far from being problematic, these different ideological conceptions of ordinary democratic politics play a crucial role in supporting a dynamic political constitution.

This article casts fresh critical light on ongoing debates about political constitutionalism. It focuses on political constitutionalist thought within British constitutional discourse. Although it is possible to detect traces of political constitutionalist thought in the United States,^2^ Canada,^3^ New Zealand,^4^ China^5^ and some South Asian states,^6^ the British tradition still dominates the literature and often inspires accounts within these states. This British tradition has been shaped around ongoing debates about the UK’s changing constitution and the dominant political ideologies within the UK’s political system. Through an interpretative inquiry, I use ideologies to uncover the logical and cultural assumptions different political constitutionalists hold about democracy, politics and constitutions.^7^

This article encourages political constitutionalists and public lawyers to critically reflect on their assumptions about political constitutionalism, its proponents and our understandings about ordinary democratic politics within constitutions. It will not analyse how ideologies inform legal constitutionalist thought nor respond to criticisms of political constitutionalists’ claims about judicial review.^8^ My goal is not to reignite the tedious political versus legal constitutionalist debate, but rather to analyse political constitutionalism on its own terms. Although juxtaposing political and legal constitutionalism might sometimes be useful in analysing constitutional change, it can also unduly inhibit how we imagine and critically reflect on each theory.

This article challenges the notion that all political constitutionalists share identical beliefs about law, government and the relationship between citizens, the legislature, the executive and the judiciary by demonstrating how political constitutionalism can appeal to different ideological traditions, often simultaneously but for distinct reasons. Moreover, the article aims to resolve the ongoing debate among political constitutionalists on whether preserving a political constitution necessitates an ideological consensus or division.^9^ I argue that ideological diversity is necessary for ensuring a dynamic and sustainable political constitution, as different ideological readings of political constitutionalism generate constitutional imagination.

This inquiry proceeds at three levels of abstraction. At the lowest level, it maps out the various works of major political constitutionalists who have significantly influenced the development of the theory.^4^ This is not intended to provide an exhaustive survey of political constitutionalist literature, as some political constitutionalists have not declared their ideological preferences, so I will not impose a label upon them. At the more intermediate level, it analyses the main ideological readings of political constitutionalist thought, showing how they articulate different understandings of ordinary democratic politics. Due to practical limits, I cannot completely explain every ideology in detail. Instead, I provide a brief best-case account of each ideology that has influenced the development of political constitutionalism. At the highest level, it reflects on the ideological conditions required to sustain a political constitution.

The article proceeds as follows. Section 2 reflects the nature of political constitutionalism as normative political theory and its relationship with ideology. Sections 3–6 present the four main readings of political constitutionalism—democratic socialist, liberal, republican and conservative—and how they understand ordinary democratic politics. Section 7 posits why a healthy ideological division is necessary to perpetuate a political constitution.

## 2. Political Constitutionalism as a ‘Thin’ Constitutional Ideology

### A. The Central Case of Political Constitutionalism

There are four commonly held tenets in political constitutionalist thought.^10^ These tenets reflect the significant and overlapping ideas that pervade the existing literature to a high degree and allow us to identify central and peripheral examples of political constitutionalist thought. These are used in the following sections to map how each ideological reading provides a different interpretation of political constitutionalism. Political constitutionalist thought can be identified by the presence of all four of the following interrelated beliefs:

I. The recognition that there will be reasonable disagreement within a political community about a course of action, rights, policy or even the constitution itself, including any protection it gives to fundamental rights.
II. The belief that reasonable disagreement should be managed by ordinary democratic processes and political institutions. This should include free, fair, competitive and regular democratic elections, as well as democratic representative and accountable political institutions. The ordinary democratic process should promote procedural norms of political equality, majority rule and contestability (expressed as the idea of being able to promote change and accountability)

Most modern constitutional theories share these first two beliefs. However, constitutional democracies must constantly balance the tension between individual rights and popular sovereignty.^11^ Political constitutionalism diverges from other constitutional theories by tilting towards popular sovereignty:

III. The belief that the constitution should not (or only sparingly) remove political decisions from the reach of the ordinary and representative democratic processes and political institutions. The constitution should be ‘what happens’ in the management of reasonable disagreement by ordinary democratic processes.
IV. The belief that laws and, by extension, judicial institutions are tools of and should faithfully work to uphold the intentions of the democratic legislature in the management of reasonable disagreement. Judicial institutions should perform a modest and complementary role, resolving legal disputes and preventing the executive branch from transgressing the statutory powers conferred on it by the legislature.^12^ Strong form judicial review is rejected.^13^

There are three important things to note about political constitutionalism thought. First, political constitutionalist thought must embrace all four tenets to a high degree at both a surface and deeper conceptual level. At the surface level, all four tenets are necessary to distinguish political constitutionalism from other constitutional theories. For example, other constitutional theories may recognise reasonable disagreement (I) and the importance of ordinary democratic processes (II) but reject (III) and (IV) by making claims about how the courts should have the final say on the interpretation of some constitutional questions.^14^ At the deeper conceptual level, these tenets need to be not only present, but also arranged in a logically coherent manner. Political constitutionalist thought has an internal logic that transforms these tenets into a coherent and distinct constitutional ideology. This also allows one to differentiate between central and peripheral cases of political constitutionalist thought.

The internal logic of political constitutionalist thought recognises that political communities are subject to persistent disagreement about how they should be governed (I). It sees reasonable disagreement as both a present and potential problem, as any decision taken in the present may become a source of reasonable disagreement in the future.^15^ However, a political community without the means of managing disagreement risks paralysis or disintegration. Political constitutionalists believe that the legitimacy and authority of a constitution depend on its alignment with three procedural norms: *political equality*, *majority rule* and *contestability* (as a means of ensuring change and accountability).^16^ They posit that each of these norms is best actualised by the ordinary political process through democratic elections and institutions (II). Accordingly, political equality, majority rule and contestability are the procedural core that undergirds any political constitutionalist commitment to the ordinary democratic process. An entrenched constitution guarded by the courts may jeopardise political equality and contestability by privileging the beliefs of the drafters and judges over those of the rest of the political community (III and IV). Thus, political constitutionalists prefer ordinary democratic processes for managing reasonable disagreements, as they provide equalitarian, majoritarian and accountable decision making (II).

In central cases of political constitutionalist thought, these tenets are present to a high degree and are coherent with this internal logic. In peripheral cases, these tenets are present but deployed incoherently. For example, political constitutionalism has been used to defend the constitutional reforms implemented by the Fidesz government in Hungary and the Law and Justice (PiS) government in Poland.^17^ While both appear to make political constitutionalist-style claims about popular sovereignty and majoritarianism to legitimise their respective reforms, on closer examination, it is clear that their reforms are designed to convert Fidesz and PiS’s transitory electoral majority into a legally authorised permanent majority.^18^ This contradicts the internal logic of political constitutionalism by rejecting the idea of reasonable disagreement as a present and potential issue by seeking to shield their respective governments from being challenged or held to account through both political and legal processes in the future. Such arguments are peripheral (and abusive) cases of political constitutionalist thought.

Second, political constitutionalists usually self-consciously aspire to prescribe as minimally as possible how a constitution should operate, at least compared to some other constitutional theories.^19^ Reasonable disagreement’s role within the internal logic of political constitutionalist thought posits that the legitimacy and authority of a constitution should not be determined by substantive moral outcomes. As Graham Gee and Grégoire Webber highlight, political constitutionalism seems to direct ‘political actors to design an electoral process based on some notion of equal votes and to ensure that the political process is based on some notion of holding those in power to account’.^20^ This is intentionally a minimalistic prescriptive pattern of constitutional thought centred around ordinary democratic politics.

Third, despite the centrality of ordinary democratic politics within political constitutionalist thought, political constitutionalism does not, at least explicitly, appear to reflect a specific understanding or normative preference about how ordinary democratic politics ought to operate. We can assume that political constitutionalists see the ordinary democratic process as synonymous with representative democracy.^21^ However, representative democracy is as contested as ordinary democratic politics. On the surface, political constitutionalist understandings of democratic politics often appear vague. For example, it is unclear whether the function of law in the management of reasonable disagreement is to promote liberty through limited interference or through exerting greater control over economic and social life. Similarly, should government be strong or limited? Should the legislature or the executive be the dominant partner in the law-making process? By what standards and means should political actors be held to account? Each of these issues, and others, can profoundly impact inter- and intra-institutional arrangements that decide how ordinary democratic politics operates within a political constitution. My claim is that political constitutionalists disagree on these questions in large measure because of their ideological understandings of how the ordinary democratic process should work.

### B. Thin and Thick Ideologies

What is meant by the ordinary democratic process remains open to interpretation within political constitutionalist thought. This omission appears to be a puzzling oversight on the part of political constitutionalists since what constitutes an ordinary democratic process is both contested and pivotal to actualising and sustaining a political constitution. Yet, on a closer reading, this omission may reflect an attempt to accommodate disagreement about the nature and value of democracy within political constitutionalist thought. Democracy and the processes used to actualise it are not easily susceptible to a singular definition, for the very idea of democracy is subject to reasonable disagreement.^22^ In juristic terms, democracy is, like justice, a *concept* about the idea of rule by the people, and there are competing *conceptions* of what a concept should entail.^23^ Political constitutionalists can rally around the concept of the ordinary democratic process so long as questions about its precise role and functioning are left open to each to interpret.

Political constitutionalism should be understood as a thin constitutional ideology. Whilst it may serve to influence the behaviour of constitutional actors,^24^ its ability to do so effectively is limited because of its minimalistic nature. As a thin ideology, it cannot offer any practical directions or answers to practical political questions, such as the nature of ordinary democracy, without the aid of thicker political ideologies, such as socialism, liberalism, republicanism and conservativism, which have long developed to provide political actors with potential responses to such questions.^25^ Consequently, what is meant by the ordinary democratic process is constituted in part by the thick ideology that one subscribes to.

Political constitutionalism was never grounded in one specific conception of democracy. Over time, different proponents have justified political constitutionalism through their preferred ideological conceptions of democracy, whether that be through democratic socialism, liberalism, republicanism or conservativism. Thus, there are different ideological readings of political constitutionalist thought. The relationship between these ideological readings and the overview presented above resembles a Venn diagram. Each reading overlaps with the others to a degree, forming the meta but ultimately thin constitutional ideology of political constitutionalism. Yet, each reading is subtly different from the others because their respective thick ideologies supply more specific meanings to concepts within political constitutionalist thought, such as how the ordinary democratic process should operate.

### C. What Are Ideologies?

It is important to clarify what an ideology means and its functions in political discourse, as the term is subject to various competing definitions.^26^ Without clarifying what ideology means, we risk talking past one another. This is particularly necessary within the context of legal theory, as ideology has mostly been treated with disdain.^27^ This article relies on an understanding of ideologies as patterns of political concepts that structure political life, enabling political actors to interpret and attribute specific meanings to political facts and events.^28^ This understanding is advanced by the prominent political theorist Michael Freeden, who posits that ideologies perform two interrelated functions.

First, ideologies enable intensely contested political concepts, such as democracy, liberty and justice, to become relatively compatible within specific ideological patterns or conceptions.^29^ This is achieved by grouping and linking different contested concepts until they acquire a relatively de-contested meaning within each ideology. This enables groups of political actors to adopt a shared conception of the concept, and inter-ideological disagreement is minimised, allowing political energies to be more directed towards the *ideology A* versus *ideology B* level of disagreement about what should be the socially legitimate interpretation of the concept. Second, by de-contesting concepts, ideologies reduce the inevitable variety of potential and conflicting understandings into more manageable ideological sets to guide political actions.^30^ Accordingly, the interpretative study of ideology is concerned with mapping out those collectively held beliefs among different political groups that enable them to understand their world and their place within it.

Just as words only have meanings in relation to their place within a sentence, concepts acquire meaning from their place within a specific pattern.^31^ An ideology is a pattern of core, adjacent and peripheral concepts that acquire specific meanings based on their proximity to other concepts within the pattern.^32^ For example, liberty is usually recognised as the core of liberalism, with democracy, human rights and equality as adjacent and less important concepts at the periphery.^33^ Although these concepts may appear across multiple ideologies, their meaning changes based on their relationship with other concepts within each ideological pattern.

Nevertheless, not every pattern of concepts can be possible due to logical and cultural constraints.^34^ Logic may impose constraints on which concepts are compatible with one another. For example, liberty and autonomy are logically compatible, but equality and racism are incompatible with one another. Similarly, culture imposes temporal and spatial constraints on the possible arrangement of concepts within an ideological pattern. Since ideologies aim to tie political thought to political action, cultural constraints anchor ideologies to material conditions. It might be logical to advocate low taxation, but it might be culturally incompatible in societies and time periods where voters universally accept paying higher taxes in return for higher quality public services.

This introduces further considerations for identifying central and peripheral cases of political constitutionalist thought and their relationship with certain ideologies. First, political constitutionalism may not be logically compatible with every thick ideology. Logic may prevent the thick ideology from fleshing out political constitutionalism’s understanding of ordinary democracy. For example, a fascist embrace of political constitutionalism would be logically incoherent, as the former is opposed to the democratic, equalitarian and pluralist values of the latter. Second, the compatibility with a thick ideology is also conditional on temporal and spatial conditions. Political constitutionalism may be compatible with democratic socialism in the UK but be incompatible with democratic socialism in another state with a very different political and constitutional culture. Additionally, ideological compatibility may change over time as cultural and political circumstances evolve. A central case of ideological political constitutionalist thought is identifiable when there is sufficient conformity with the inner logic of political constitutionalism and when political constitutionalism is logically and culturally compatible with the thick ideology.

Finally, I am not suggesting that these ideological readings of political constitutionalism reflect the official policy positions of any of the major political parties in the UK. Indeed, some of the ideological readings discussed below currently lack a partisan vehicle for pursuing their vision. This is not problematic as ideological thought is articulated across a spectrum of various sources, including the works of philosophers, politicians, think tanks, scribes and agitators. Most political constitutionalists have been influenced or aim to present their claims as philosophical rather than overtly partisan claims. Nevertheless, whether one presents their claims in a philosophical or partisan manner, their claims reflect ideological patterns of political thought that can be subject to interpretative analysis.

### D. Summary

To summarise, we should understand political constitutionalism as a thin constitutional ideology that relies on thicker political ideologies to flesh out what is meant by ordinary democratic politics. Although all political constitutionalists may collectively unite around a minimalist set of concepts and beliefs, individual (or clusters of) political constitutionalists have inadvertently, perhaps deliberately, assigned more precise meanings to these values and ideas by viewing them through the lens of their respective ideologies. Since values and ideas acquire different meanings through different ideologies, it becomes possible to delineate between different readings of political constitutionalism within the existing literature. The extent to which the values and ideas of political constitutionalism can fit with an ideology is conditioned by the logical and cultural constraints surrounding each respective ideology. Consequently, political constitutionalism may not be compatible with or appeal to every ideology.

## 3. Democratic Socialism

There is a long-assumed affinity between British democratic socialism and political constitutionalism.^35^ Yet, there are no obvious authoritative writers or works that completely encapsulate the democratic socialist reading of political constitutionalism. This is because only aspects in the legal scholarship of John Griffith, Keith Ewing, Conor Gearty and Danny Nicol, among others, suggest sympathy towards democratic socialist and political constitutionalist ideas. To various degrees, each has sought to locate themselves within and continue a long-held tradition of democratic socialist constitutional thought. Their claims are usually implicit, serving as a conceptual underpinning for their critiques of aspects of public law, labour law and economic change.

Understanding the democratic socialist reading requires looking beyond both the thinly normative claims that underpin John Griffith’s functionalist account of the constitution within his famous lecture on ‘The Political Constitution’^36^ and those explicitly normative claims that emerged following the theoretical turn in public law thought, such as those found within Keith Ewing’s ‘The Resilience of the Political Constitution’.^37^ One must instead look to those early 20th-century democratic socialists who influenced these scholars. Though many democratic socialists never expressly spoke of political constitutionalism, many of their claims about the normative desirable nature of the UK’s constitutional arrangements chime with the modern theory.^38^ Consequently, the development of the democratic socialist reading has been heavily culturally conditioned by the political, economic and constitutional conditions of the UK.

### A. Socialism, Freedom and Democracy

To briefly summarise, socialism is about the promotion of greater economic, social and political equality.^39^ It intertwines equality with the concept of liberty, resulting in a positive understanding as ‘the ability to act, not merely to resist’.^40^ Collectivism reflects socialism’s emphasis on the community, rather than the individual, as the central principle of social organisation.^41^ Accordingly, socialism favours a more collectivist administrative state, promoting fraternity, personal service and ‘the subordination of individual ends to the common good’.^42^

Gradualism reflects a socialistic faith that historical progress will inevitably lead a society to transition away from capitalism.^43^ The revisionist and gradualist traditions posit that change should come through democratic means and view the state as an instrument that can be deployed towards both capitalist and socialist ends.^44^ Crucially, gradualism allows democratic socialists to favour the open nature of the UK’s constitution.^45^ As Sidney Webb observed, ‘in no other country are statesmen so ready as in England to carry out political proposals pressed upon them from below … the gradual “socialising of politics”… is rendered possible by the fluidity of the English Constitution’.^46^

The German social democrat Eduard Bernstein’s contribution is especially important in connecting socialism and democracy and, in turn, democratic socialism to political constitutionalism.^47^ Bernstein understood democracy as a means of managing reasonable disagreement between different social classes within a political community. Bernstein described democracy as ‘the absence of class government … a state of society in which no class has political privilege which is opposed to the community as a whole’.^48^ Although democracy could not eliminate social class, Bernstein believed it could provide the means of managing disagreement in a way that promoted tolerance and co-operation between classes.^49^ Democracy could foster socialism via the right to vote, incentivising individuals to co-operate with others within their community.^50^

Crucially, Bernstein makes democracy a logical constraint on socialism, creating democratic socialism, as democracy was not a purely instrumental means of bringing about socialist change but is also an end in itself.^51^Bernstein cast democracy and socialism as inseparable, arguing ‘to betray democracy is to betray socialism’.^52^ Thus, a democratic socialist government must tolerate dissent to avoid betraying its own ideology.^53^ A democratic socialist form of political constitutionalism recognises and tolerates reasonable disagreement between those who wish to preserve the status quo and those who wish to bring about change.

### B. A (Socialist) Government that Can Govern

It is possible to see how a democratic socialist reading of political constitutionalist thought emerges. Democratic socialists recognise reasonable disagreement as the product of class division and between those who support and oppose socialist change (I). Democratic socialists perceived that the UK constitution serves the interests of the class which controls the state. The constitution may function negatively to preserve the status quo or positively to bring about socialist change.^54^ Democratic socialists concluded that ordinary democratic politics is key to promoting socialism in at least four ways (II). First, democracy provided a peaceful means of managing class conflict.^55^ Second, democracy had rendered capitalism vulnerable to being contested; therefore, democracy ensured capitalism could be replaced by socialism.^56^ Third, if the constitution was a tool of the dominant class and democracy allowed for a peaceful transition between the classes, a democratic form of constitutionalism could promote socialism.^57^ Democratic socialists were confident they could muster a majority in support of a socialist government. Fourth, representative democracy ensures that citizens, regardless of their class, will have relatively equal opportunities to influence decision making, actively participate in political fraternities and perform their functions by standing for political office.^58^

A preference for political constitutionalism can also be inferred from democratic socialists’ concerns about constitutional obstructionism. Democratic socialists were largely apathetic towards the idea of constitutional entrenchment (III). Democratic socialism in the UK was culturally influenced by the uncodified nature of the UK constitution. Stafford Cripps described the absence of an entrenched constitution as ‘our greatest asset, it should enable the constitution to adapt itself momentarily to the desires and wishes of the people’.^59^ The democratic socialist reading recognises that the legal principle of parliamentary sovereignty creates the potential for a radical socialist transformation of society and simultaneously prevents capitalist elements from directly challenging or frustrating socialism.^60^ Moreover, British socialists were generally apathetic about questions of constitutional reform since they were primarily focused on questions of economic governance.^61^

Democratic socialists have often viewed the judiciary as a potential tool of obstructionism by capitalist interests (IV). They have long rejected the belief that judicial decision making is an apolitical activity and that judges are neutral arbiters.^62^ The common law is perceived as historically being ideologically committed to the ideas of individual economic liberty and the defence of private property.^63^ This scepticism is partly a product of the cultural conditions in which democratic socialist thought emerged at the turn of the 20th century. Controversial cases such as *Taff Vale Railway Co v Amalgamated Society of Railway Servants*,^64^*Amalgamated Society of Railway Servants v Osborne*^65^ and *Robert v Hopwood*^66^ fuelled concerns that the judiciary could be deployed to frustrate socialism.^67^ Scepticism towards the judiciary also appears as a logical constraint within socialist thought, for socialism is about bringing forward change and, as Griffith argued, the judiciary naturally functions to ‘preserve and to protect the existing order’, creating fertile scope for disagreement between the two.^68^

The democratic socialist reading of political constitutionalist thought sees ordinary democracy as promoting strong government. This is reflected in both their scepticism towards constitutional obstructionism and their commitment to change. On the one hand, obstructionism is viewed as distorting the accountability of the government to the people.^69^ Obstructionist tactics are described as ‘denying the will of the people and hindering a democratically elected government’.^70^ The governing party is rendered accountable to the electorate, based upon the extent to which it delivers its electoral promises and the consequences of its policies. Moreover, democratic socialists are keen to bring about change as quickly as possible once in government. Accordingly, democratic socialist political constitutionalists emphasise that the constitution should function as ‘a lubricant, rather than a barrier to social, economic and political change’.^71^ Any reforms that may endanger strong government are traditionally viewed as a threat.

## 4. Liberalism

The liberal reading of political constitutionalism is found almost exclusively in the works of Jeremy Waldron.^72^ Nevertheless, Waldron’s contribution to political constitutionalism is unusual in that Waldron is not British, nor does he use the term ‘political constitutionalism’ or directly engage with other political constitutionalists’ arguments within his work. Nevertheless, his work has had a remarkable influence in the development of political constitutionalism. Indeed, Aileen Kavanagh has gone as far as describing Waldron as the high priest of political constitutionalism.^73^ This is due to Waldron’s timely contributions to constitutional theory, which coincided with the enactment of the Human Rights Act 1998.^74^ At a time when many had grown frustrated with the limits of Griffith’s functionalist defence of the UK political constitution, Waldron’s explicitly normative defence of ordinary democratic politics against liberal legalism was welcomed, but also allowed for the rejuvenation of political constitutionalist thought.^75^ Across various works, Waldron has sought to reconceptualise liberal constitutionalism in a way that closely aligns with political constitutionalism.

### A. Waldron’s Liberal Freedom

Waldron offers his own conception of liberalism that prioritises democracy as the central means of protecting liberty. For Waldron, liberalism demands a society

chosen by the people living under it, something whose main features are as intelligible to them as the charter of a club of which they are founding members, designed by them to serve the purpose that bought them together in the first place.^76^

Under this conception, liberty is undermined by *imposed rules*, which lack the consent of the individual and therefore fail to recognise ‘the capacity of human agents to determine for themselves how they will restrain their conduct’.^77^ In contrast, liberty is promoted through *consented rules*, which are restraints that the individual consents to. Crucially, like most other social contract theorists, Waldron accepts this consent can be actual or hypothetical in form.^78^

Reasonable disagreement emerges from each and every individual’s capacity to engage in autonomous moral reasoning (I). Individual autonomy and social interdependency give rise to what Waldron calls ‘the circumstances of politics’, which is the ‘felt need among the members of a certain group for a common framework or decision or course of action on some matter, even in the face of disagreement about what that framework, decision or action should be’.^79^ For Waldron, this must be managed in ways that respect individual autonomy by ensuring every individual within a political community has the right to participate in the decision-making process.^80^ As Waldron reasons, ‘it is precisely because I see each person as a potential moral agent, endowed with dignity and autonomy, that I am willing to entrust the people en-masse with the burdens of self-governance’.^81^ Accordingly, self-governance is presented as both a means of self-realisation and a recognition of social interdependency.

Waldron concludes that ordinary democratic politics can offer an effective and legitimate method of managing the circumstances of politics that respects human autonomy (II). Ordinary democratic politics can broadly be understood as a decision-making process that combines a representative legislature and electoral politics.^82^ Waldron’s account tends to focus almost exclusively on the role of a representative legislature in managing reasonable disagreement. Legislative decision making is said to reflect the notion that if a decision must be taken ‘by some institution which comprises fewer than’ the entire population, that institution should by design be diverse, pluralistic and accountable, to embody ‘the spirit of self-government, a body in which we can discern the manifest footprints of our own original consent’.^83^

From here, Waldron contrasts legislative institutions against constitutional entrenchment and judicial institutions. Legislatures are understood as public, pluralistic, diverse deliberative bodies which purposively make and amend law, in ways that respect and ensure equal decision-making weight to the views of each and every individual (or at least their elected representatives) within a political community.^84^ In contrast, an entrenched constitution, guarded through judicial institutions, becomes viewed as creating imposed rules. Waldron considers constitutional entrenchment as the ‘artificially sustained ascendancy of one view within the polity over other views’ within a political community (III).^85^ Entrenchment is seen to be premised on the misguided assumptions that the potential reasoning used by future generations will always be flawed.^86^ Similarly, when judges engage in political decision making via strong-form judicial review, they are creating imposed rules (IV). Waldron argues that the autonomy of each and every individual ‘is lost when an unelected and unaccountable individual or institution makes a binding decision’ that overrules the reasoning of citizens and their representatives.^87^ For a liberal political constitutionalist, legislation is an achievement of autonomous reasoning and social interdependence in the circumstances of politics; accordingly, respect for legislation is owed by both ordinary citizens and judges.^88^

### B. Limited Government

In comparison to the other readings of political constitutionalist thought, the liberal reading has developed a more concrete conception of ordinary democratic politics. In *Law and Disagreement*, Waldron presents the ordinary democratic process in an extremely abstract and idealised way.^89^ It is largely silent on how institutional arrangements, political parties and elections might work. As a result, one may conclude the liberal reading of political constitutionalism can accommodate limited and interventionist styles of governance within its conception of ordinary democratic politics.

However, there is a more pronounced emphasis on a proceduralist conception of deliberative democracy and limited government within Waldron’s more recent *Political Political Theory.*^90^ Here, Waldron covers a wide range of issues, including the nature of constitutionalism, the separation of powers and judicial review, but it is in his considerations about the legislature’s role where the liberal political constitutionalist account of ordinary politics is most vibrant. Waldron argues that a healthy democratic legislature must promote a range of principles, including transparency, diversity, respect for opposition, procedural equality, majority rule, a commitment to being governed by clear procedural rules, a duty of care and responsive deliberation.^91^ By requiring a legislature to have a duty of care towards citizens and engage in responsive deliberation, this liberal reading gives rise to the idea of promoting limited government through ordinary democratic politics.

The idea of limited government is a common feature of liberal ideologies. The idea of institutionalising a duty of care among legislators is considered necessary to avoid legislation, intentionally or unintentionally, unduly restricting the liberty of individuals.^92^ Although liberal political constitutionalists will believe that a democratic legislature should be endowed with the legal principle of legal sovereignty, they will emphasise that this power must be exercised responsibly to avoid creating imposed rules. For Waldron, this is best achieved through slowing down and layering the decision-making process.^93^ This is realised by ensuring the decision-making process incorporates several types of debates. At a minimum, we should aim to debate the issue first with passion and excitement, then again with thoughtfulness and prudence.^94^ The ordinary democratic process must provide different layers of decision making with various stages, such as the election and then the various stages within the legislative process.^95^ Waldron views responsive deliberation as the requirement that legislators do not enter debates with fixed preferences and must be open to the possibility of changing their minds when confronted by the force of (albeit vaguely defined) superior reasoning and argument within the legislative debates.^96^

When a legislative duty of care and responsive deliberation interact, one can see how the liberal reading of political constitutionalism promotes a limited government vision of ordinary democracy. For a liberal political constitutionalist, the democratic legislature functions to temper the perceived excesses of electoral democracy in two senses. First, if our democratic process is layered, the outcome of an electoral decision is insufficient on its own to justify legislation placing conditions upon human autonomy. For a liberal political constitutionalist, an election is simply one decision-making process out of many within the ordinary democratic process. Second, responsive deliberation within the legislature requires legislators to function as trustees, rather than delegates. If legislators were delegates, they would be unable to yield to superior arguments without betraying their electoral mandate. Accordingly, it is the final decision of the democratic legislature which decides how legislation manages the circumstances of politics and imposes conditions upon individuals. A liberal political constitutionalist will not view legislative supremacy as synonymous with popular sovereignty; rather, legislative sovereignty functions as a means for securing a liberal commitment to limited government.

## 5. Republicanism

The republican reading of political constitutionalism is primarily articulated in the works of Richard Bellamy and Adam Tomkins.^97^ Republicanism has often been presented as an alternative to liberalism.^98^ This is because both ideologies emphasise similar core concepts, such as liberty and self-mastery. Whereas liberalism understands liberty as freedom from interference, republicanism conceives liberty as *freedom via non-domination*.^99^ Republican political constitutionalist thought distinguishes itself from the liberal reading, through its belief that the avoidance of domination requires prioritising ordinary democracy and the rejection of entrenchment and strong-form judicial review. Yet, the republican and liberal readings of political constitutionalism share similarities.

### A. Freedom via Non-domination

In republican thought, domination occurs when an actor, group or institution wields power over others, in the sense that they have the capacity to interfere with others on an *arbitrary basis*.^100^ Crucially, domination can occur in a procedural sense, as the simple possibility of interference signals domination.^101^ Freedom via non-domination reflects the idea that interference may not always mean domination if the interfering actor is obliged to ‘track the interests and ideas of the person suffering the interference’.^102^ The arbitrary exercise of power can be avoided in two ways: first, the exercise of power can be subject to preconditions which filter out what the affected would consider unsuitable behaviour;^103^ and second, arbitrary interferences can be subject to sanctions.^104^ In essence, avoiding domination requires the powerholder to be made accountable for their actions. A significant benefit of freedom being understood through the lens of non-domination is that, in the context of the modern state, it allows some forms of interference by the state to be legitimate.^105^

For the republican reading, freedom via non-domination shapes how the circumstances of politics should be managed. Like the liberal reading, the republican reading recognises that human reasoning will give rise to disagreement on matters that require collective action (I).^106^ In response, both Bellamy and Tomkins contend that reasonable disagreement must be managed in ways that promote freedom via non-domination. Both emphasise that reasonable disagreement should be managed through the ordinary democratic process to ensure interference by the state is non-arbitrary (II). Nevertheless, Bellamy and Tomkins draw upon different aspects to support this claim.

### B. *Non-domination by* Audi Alteram Partem *or Throwing the Scoundrels Out?*

For Bellamy, the ordinary democratic process provides a normatively attractive decision-making process that avoids domination by ensuring relatively equal decision-making weight among citizens. The avoidance of domination requires a decision-making process that promotes equal respect and concern towards citizens and does not privilege certain views as superior.^107^ For Bellamy, ordinary democratic politics can promote this in three senses. First, the ordinary democratic process avoids domination by treating and respecting each citizen’s view with equal weight in the decision-making process.^108^ Second, majoritarian decision-making processes provide a practical, statistical and neutral method of collective decision making.^109^ Third, Bellamy views ordinary democratic processes as promoting an organic balance of power between rival political parties.^110^ The organic balance of power incentivises different groups and political actors to ‘hear the other side’ during the decision-making process.^111^ As different political rivals compete to form a majority, they are incentivised to engage with different political views in a respectful manner, as they may need to incorporate these opinions into their own policy platforms in the future. Building a winning majority requires accommodating and promoting compromise between competing views.^112^ In doing so, rival parties are incentivised to monitor each other’s behaviour, with those out of office having a powerful incentive to hold the governing party accountable.^113^

In contrast, Tomkins’ account places a stronger emphasis on how ordinary democracy promotes accountability to avoid domination. Unlike Bellamy, who understands accountability as being achieved through processes that force rival political parties to hold each other to account, Tomkins eschews the language of hearing the other side and sees political parties as a threat to accountability. Instead, Tomkins contends that domination is best avoided by ensuring that those who exercise the political power to manage disagreement are rendered accountable to the citizens of the political community because such power is exercised upon them and in their name.^114^ The implication of this is a stronger emphasis on sanctioning political behaviour through electoral and parliamentary procedures. The closest Tomkins gets to implying the need to hear the other side is in his suggestion that the contestability of government policy via electoral and parliamentary accountability mechanisms can incentivise the government to track the interests of those who would be affected by the exercise of the state’s power.^115^ In essence, those who govern must hear the governed. Nevertheless, Tomkins’ arguments are heavily culturally conditioned in that his objective is to position the concept of political accountability as the normative foundation of the UK’s predominantly political constitution. Consequently, domination is avoided by ensuring governments are held to account by Parliament, particularly through their requirement to maintain the confidence of the House of Commons.^116^ Similarly, Members of Parliament are made accountable for their behaviour to the electorate through elections.^117^

The republican reading’s emphasis on freedom via non-domination also results in a wariness towards constitutional entrenchment and strong-form judicial review (III and IV). Once again, Bellamy and Tomkins offer different justifications for their distrust. In Bellamy’s case, distrust stems from his belief that attempts to de-politicise certain issues through entrenchment and judicial decision making risk creating domination. In the circumstances of politics, to demarcate ahead of time certain rights or rules as beyond disagreement or to insist that the interpretation of these rights and rules should be decided by non-political forums creates domination by privileging the views of some citizens over the rest of their political community.^118^ In response, Bellamy contends that domination is avoided when a constitution allows citizens to self-govern their community. Therefore, the constitution should be organised around the idea that the ‘democratic process is the constitution’.^119^ Similar to his arguments about accountability, Tomkins’ distrust of constitutional entrenchment and strong-form judicial review is informed by cultural influences. His rejection of constitutional entrenchment reflects a general confidence in how the UK’s unwritten constitution has functioned.^120^ An entrenched constitution is simply unnecessary because the UK’s constitution is perceived to be held together by the constitutional convention that the government of the day must retain the confidence of the House of Commons to remain in office.^121^ Moreover, Tomkins is often pessimistic about the capacity of judges to uphold a bill of rights and provide effective legal accountability against the executive.^122^ Legal methods of accountability are considered to be constrained due to the nature of legal decision-making processes. For Tomkins, because judges are reactive, confined to legal argument and ill-equipped to engage in policy making, legal accountability is ‘always haphazard, not systemic’ in design.^123^ Finally, Tomkins also argues that strong-form judicial review can promote domination because judges are undemocratic, which prevents citizens from easily contesting their reasoning, at least in comparison to their elected representatives.^124^

The republican reading of political constitutionalist thought arguably comes the closest to presenting a true minimalist vision of ordinary democracy. For example, Bellamy understands the ordinary democratic process as a never-ending and self-sustaining electoral cycle in which rival aspirants can compete and hold each other to account. Bellamy is often neutral on the function of government and the function of law. Nevertheless, Bellamy briefly considers whether the relationship between citizens and their representatives should take a trustee or delegate form. At one point, Bellamy briefly but explicitly remarks ‘representatives must regard themselves more like mandated delegates than as trustees’ in that their task is to represent and implement the electorate’s wishes.^125^ One may infer from this that Bellamy understands electoral politics as an agenda-setting decision-making process and legislative decision making as the means of implementing said outcomes.

In contrast, Tomkins’ conception of ordinary democracy as a means of promoting effective political accountability and the cultural influence of the Westminster Parliament favour a more limited conception of government. Although Tomkins acknowledges that citizens have a role to play in promoting the common good, he tends to cast Parliament as an enlightened guardian of the people.^126^ His argument for limited government is most pronounced in his criticism of party politics and executive dominance at Westminster. Tomkins’ central concern is that the nature of partisan politics results in parliamentarians who will prioritise their party’s interests over the public’s, which often benefits the government. As Tomkins contends, ‘if the government can control the Commons, then clearly the ability of the Commons to freely and fully scrutinise the government and to hold it to account is in danger of being compromised’.^127^ To remedy this problem, Tomkins has considered it necessary to prohibit party whips.^128^ This results in an important difference between Bellamy and Tomkins. For Bellamy, political parties are central to avoiding domination as they promote the need to hear the other side. In contrast, for Tomkins, parties distort the accountability mechanisms.

## 6. Conservativism

The latest reading of political constitutionalism to emerge articulates a conservative reading. This has primarily been articulated by Graham Gee^129^ and, to a lesser extent, in some works by Richard Ekins^130^ and Jonathan Sumption.^131^ There has been growing interest in political constitutionalism among the political right in recent years.^132^ At least within the British context, this may appear to mark a drastic change in constitutional thinking among conservatives, as historically conservative readings of the constitution often looked positively upon the common law and the judiciary as repositories of conservative reasoning that tempered the excesses of democratic government.^133^ This is challenged by Gee’s claim that a conservative reading of political constitutionalist thought has long coexisted alongside the democratic socialist reading discussed above.^134^ For Gee, the appeal of political constitutionalism rests in its capacity to preserve and perpetuate a perceived political heritage of strong but limited government and an organic social order against certain types of change that are premised upon abstract reasoning.^135^ To render explicit why the political model appeals to conservativism, Gee draws upon the works of Michael Oakeshott, John Kekes and Anthony Quinton, among others, to highlight four concepts of conservative thought that flow from the need to control change: *traditionalism*, *organicism*, *scepticism* and *human imperfection*.^136^ Crucially, the conservative reading diverges from the other readings in some aspects because of the distinctive nature of conservative ideology.

### A. Conservativism as a Sui Generis Ideology

Conservativism is a complex and unique ideology that distinguishes itself from other rival ideologies in two senses. First, conservativism eschews the inclusion of abstract political concepts, such as freedom or equality. Second, conservativism often seeks to present itself in opposition to whatever other ideologies it correctly or incorrectly perceives as threatening the existing order.^137^ This gives conservativism a mirror-like quality; it routinely changes to meet the perceived needs of the day. Fundamentally, conservativism is an ideology that is ‘concerned with the problem of change: not necessarily proposing to eliminate it, but to render it safe’.^138^ It is concerned with protecting common goods from the threat of reckless change.^139^

Traditionalism can be, albeit loosely, understood as a preference for the established over the potential, the known over the unknown.^140^ What is or could be considered a tradition is purposefully left vague by conservatives. There is no authoritative list of traditions that conservatives wish to defend. Crucially, conservatives accept that traditions are not static; different traditions continuously evolve through interactions with one another and new customs, beliefs and practices that result from the changing world.^141^ Edmund Burke contended that ‘a state without the means of some change is without the means of its conservation’.^142^ Reasonable disagreement emerges from the need to decide which traditions are good, tolerable or unacceptable (I). For conservatives, this responsibility ought to rest with those legitimately empowered by the political arrangements of the state and those capable of reflecting on the history of their community to decide.^143^

Reasonable disagreement can also be understood in a second sense, as the division between organic and artificially driven change (I).^144^ This division is shaped by the relationship between organicism, scepticism and human imperfection. Organicism reflects a preference for natural and gradual changes driven by social forces within a political community.^145^ Conservativism is wary of artificially driven change, which seeks to implement mechanical and experimental changes upon the community.^146^ Scepticism reflects conservativism’s belief that human intellectual reasoning is imperfect and pales in comparison to the accrued wisdom found within traditions.^147^ On this understanding, rationalistic, abstract and metaphysical ideas about the ends of politics should be avoided as they struggle to account for the subtleties and complexities of actual political practice.^148^

It is important to recognise that reasonable disagreement about traditions does not automatically result in a preference for a political constitution. It is only possible for a conservative to be committed to the arrangements of a political constitution if those arrangements have been historically successful for the political community. It is also equally possible for a conservative to be reverent of a written constitution, guarded through strong-form judicial review, if those arrangements have been historically successful within a political community. Accordingly, the emergence of a conservative reading of political constitutionalist thought is culturally constrained by the pre-existing nature of a constitutional order. Political constitutionalism can only appeal to a conservative raised within an existing constitutional tradition that closely aligns with the ideas of political constitutionalism, such as the UK (II, III and IV).^149^ Organicism allows conservatives to share in seeing the appeal of the political constitutionalist idea of the constitution as a reflection of ‘what happens’ in the management of reasonable disagreement rather than as a higher law (III). Although conservativism is not opposed to a written constitution (especially where that is a successful tradition of the community), a conservative would reject attempts to artificially impose a written constitution upon a community which has historically succeeded without one.^150^

To understand how scepticism can give rise to a conservative reading of political constitutionalist thought, we need to consider how the mirror-image nature of conservativism and the ambiguous nature of tradition versus change intersect with one another within conservative thought. What is perceived as a tradition and a threat are never fixed. A conservative defence of a tradition only becomes clear when there is a perception that an action, belief, practice or institution is threatened by artificially driven change. Therefore, conservative political constitutionalist thought only appears when conservatives perceive a political constitution is under attack. Therefore, conservative political constitutionalist arguments are framed almost entirely negatively. A conservative political constitutionalist will almost always adopt a defensive posture that seeks to present the arguments of those who advance change as failing to recognise the complexities of the political constitution as a tradition. In this sense, conservative political constitutionalists express their commitment to procedural norms of political equality, majority rule and contestability not as abstract ideals, but as implicit features of established constitutional traditions that promote good governance and are worth defending (II).

From here, we can begin to understand why conservative political constitutionalists have only recently taken opposition to political decision making by judges. As discussed above, democratic socialist political constitutionalists have long recognised that the courts are inevitably involved in political decision making through judicial review. Yet, conservative political constitutionalists have either implicitly presented this as a new phenomenon or tacitly admitted their silence when judicial review has been deployed by a conservative judiciary to frustrate socialist policy makers.^151^ Recent scepticism towards judicial review and the common law has only publicly appeared because conservative political constitutionalists perceive two traditions as being threatened by judicial decision making.^152^

The first is the common law as a tradition that promotes goods such as prosperity and legal certainty through adjudicating disputes between citizens and between government and citizens.^153^ This common law tradition is perceived as being jeopardised by judicial decision making that seeks to reshape the common law towards certain (non-conservative) political positions.^154^ Conservative political constitutionalists perceive this ideological change in the common law as flowing from closer European integration via the UK’s membership of the EU and the European Convention on Human Rights.^155^ In reaction, conservative political constitutionalists contend that judges should refrain from pursuing non-conservative political projects and instead focus on performing a more modest and complementary role to political institutions (IV).

### B. Strong but Limited Government

The second tradition that conservative political constitutionalists feel is threatened is the idea of a *strong but limited government*, which is considered necessary to combat human moral imperfections.^156^ On the one hand, a strong government is needed to prevent dangerous traditions from harming good traditions and protect the state from externalised threats.^157^ On the other hand, this government must be limited to prevent its own intellectual and moral imperfections from unduly interfering with and jeopardising those traditions that promote good.^158^ Consequently, for conservative political constitutionalists, parliamentary sovereignty and, by proxy, the existing institutional arrangements that allow for a high degree of executive dominance within the Westminster Parliament^159^ are perceived as valuable traditions that promote and sustain strong but limited government (II).^160^

Conservative political constitutionalist thought articulates a distinctive set of beliefs about how ordinary democratic politics should work. To begin with, it must be acknowledged that a conservative reading of political constitutionalism’s understanding of ordinary politics will be heavily informed by the localised political culture rather than prior principles about democracy. Nevertheless, it is possible to identify distinct characteristics of a conservative political constitutionalist account of democracy. As Gee acknowledges, ‘the conservative tradition of political constitutionalism unashamedly envisages an elite-led and executive-oriented constitutional order’.^161^ This reflects the general historic tendency of conservativism to view society in hierarchical terms, with rank and division being the product of the organic development of human society.^162^ This results in a meritocratic understanding of power, whereby deference is given to those who have successfully ascended to the highest offices of power.^163^ Good governance requires a sufficiently high degree of political leadership and statecraft to safely manage change within the established societal traditions.^164^ This implies that although parliamentary and intra-party scrutiny is sometimes welcome, a sufficient degree of deference should be given to the executive.^165^ This also results in a minimalist vision of democracy. Political constitutionalists have previously been criticised, sometimes unfairly, for presenting democracy as little more than a series of isolated electoral events that force voters to choose between various elites.^166^ However, conservative political constitutionalists are quite comfortable with this vision of democracy, as they embrace Joseph Schumpeter’s account of democracy as an ‘arrangement for arriving at political decisions in which individuals acquire the power to decide by means of a competitive struggle for the people’s vote’.^167^ A Schumpeterian democrat believes elections exist to produce and remove leaders and that voters should have very little influence over those leaders.^168^ This is desirable for a conservative political constitutionalist because it allows established elites to temper the democratic will of the nation to protect traditions from what they perceive as rapid and dangerous changes. This results in what Leo Amery described as ‘government of the people, for the people, but not by the people’.^169^

With its commitment to elitism and an executive-dominated vision of ordinary democratic politics, the conservative reading offers its own distinct flavour of political equality, majoritarianism and contestability. Political equality plays a less significant role when compared to other ideological readings.^170^ It exists as far as it is recognised as a successful tradition within a political community. Notions of contestability and accountability are also generally downplayed. They are viewed more as a logical necessity of organicism and human imperfection. Nevertheless, deciding the place of majoritarianism is difficult to pin down. Conservative political constitutionalists claim not to fetishise majoritarianism, yet in practice conservatives are highly dependent on majoritarianism to resist or initialise change. One might conclude that conservative political constitutionalists believe that majoritarianism is only truly acceptable under conservative governments.^171^ Consequently, a conservative reading of political constitutionalist thought appears particularly vulnerable to tilting into the kinds of inconsistencies that might render it a peripheral case.

## 7. Maintaining a Dynamic Political Constitution

As seen above, political constitutionalism can appeal to a range of political ideologies, resulting in different conceptions of ordinary democratic politics. Nevertheless, this raises the question: does a real-world political constitution require an ideological consensus or disagreement to be continued? There is disagreement among political constitutionalists on this question. One view is exemplified by Griffith, who presents the UK’s predominantly political constitution as the product of ideological disagreement.^172^ A second view is expressed by Gee, who hypothesises that a political constitution depends on a broad ideological consensus.^173^ Here, I side with Griffith, but his position needs to be expanded upon if one wishes to make a normative claim that a political constitution should recognise ideological disagreement about ordinary democratic politics. To do this, I will highlight how ideological disagreement generates the necessary constitutional imagination for sustaining a dynamic political constitution.

### A. Ideological Diversity or Ideological Consensus

Griffith’s characterisation of the British constitution emphasised ideological disagreements in two senses. First, Griffith rejected describing the constitution as equilibrium underpinned by unifying political consensus; ‘Conflict is at the heart of modern society’ because there is routine disagreement between the dominant ideological forces about governing.^174^ Second, Griffith described the constitution as a dynamic power complex. This is reflected in his claims that ‘politics is what happens in the continuance or resolution’ and ‘the constitution is no more and no less than what happens’ in the management of disagreement.^175^ The constitution is alive and may change because there are ideological disagreements.^176^ Griffith was not making a normative claim about ideological disagreement here. This was a positivist understanding of a constitution as a description of political conditions; to which Griffith highlights ideological disagreement as one important part.^177^ The lesson for political constitutionalists is that any normative understanding of a political constitution which does not recognise ideological disagreement is likely to become inconsonant with constitutional reality.

In contrast, Gee theorises that a political constitution requires a sufficiently broad and deep-rooted ideological consensus among the dominant political actors to sustain itself.^178^ This consensus sustains a political constitution by instructing political actors to recognise the political constitution as a legitimate model that they must work within and preserve. This is necessary to ensure ideological forces do not abuse their powers and to support a self-regulatory style of political discourse.^179^ Gee acknowledges that there may be dissent and conflict from time to time over some constitutional questions but is unclear about when and to what degree ideological dissent should be tolerated. As a conservative, Gee laments that there is ideological dissent about the nature of the political constitution, presenting changing attitudes as a sign of a breakdown in the supposed consensus supporting the UK’s political constitution.^180^

Gee’s hypothesis is problematic because dependence on a deep-rooted ideological consensus makes actualising and maintaining a political constitution practically difficult. An ideological consensus can only develop if the idea of the political constitution functions as a thin constitutional ideology articulating a sufficiently ambiguous set of claims capable of appealing to different ideologies simultaneously. Yet, this will become problematic when constitutional actors are confronted with real-world political issues that require them to call upon thicker ideological prescriptions to address real-world change and problems. It is here where ideological disagreement about the substantive operation of a political constitution will appear.

For example, although there are substantial differences between the democratic socialist and conservative readings of political constitutionalism, both readings are heavily animated by the question of change. The gradualist propensity of democratic socialism means its proponents broadly welcome political, social and economic changes towards socialism. In contrast, conservativism is generally wary of political, social and economic change; its proponents will defend the status quo (assuming the status quo is perceived to promote the common good). Consequently, both ideologies inform different approaches to managing reasonable disagreement and maintaining a political constitution, particularly during intense periods of political, social and economic change. Conservatives may contend that maintaining a political constitution requires defending it in its current formation. In contrast, democratic socialists may contend that maintaining a political constitution requires reform. Indeed, a cursory glance at British political history reveals routine disagreements among the governing elites about how to respond to political, social and economic changes. Consequently, an ideological consensus is unlikely to survive against the rough and tumble of real-world day-to-day politics. At best, such an ideological consensus can only exist at the highest level of abstraction, but, in doing so, it supplies little practical value to those seeking answers to real-world questions that will determine the nature of their future political constitution.

### B. Ideological Diversity as Constitutional Imagination

We should not necessarily see ideological disagreement about a political constitution as a sign of bad or waning faith. Rather, disagreement is essential to maintaining a dynamic political constitution as ideologies generate constitutional imagination.^181^ To be effective, constitutions must manage the tension between preservation and modernisation. A common method of managing this tension and generating support is by providing useful interpretative ambiguities.^182^ Through the incorporation of ambiguities, constitutions often bypass, perhaps only temporarily, difficult and divisive political issues that might frustrate their capacity to generate popular support. Constitutional ambiguities can enable a tacit agree-to-disagree attitude among the political community on such questions.^183^ Crucially, this ambiguity creates scope for political ideologies to articulate their own distinct ideological responses to those vexed political questions.

Similarly, the thin nature of political constitutionalism creates constitutional ambiguity that allows for different ideological conceptions of ordinary democratic politics to develop. Since the different ideological readings of political constitutionalism impart different conceptions of a political constitution, there can be no concrete answer to the question of whether to preserve or reform an existing political constitution. Instead, there is only reasonable disagreement. We should expect that sustaining a political constitution is decided by the ever-shifting balance of power between the competing ideological fractions within the political community.

Inevitably, this results in a dominant ideological conception of the constitution, reflecting the political ideology of the political faction that currently holds political power. The dominant ideology is deployed as both a guide and a means of legitimating how dominant political factions respond to real-world political, social and economic issues, as well as navigating the need to preserve and modernise their political constitution. This also results in rival utopian ideologies embraced by those political factions who are currently out of power.^184^ These rival utopian ideologies develop their own visions of how to preserve and sustain the political constitution. In doing so, they question the approach of the dominant ideology and may develop new innovative ideas. As Loughlin posits, constitutional imagination is borne from the combative relationship between the dominant ideology, which focuses on legitimating the constitution that exists and the utopian ideologies that seek to undermine what exists by comparing it to a normative constitutional vision.^185^ In the context of a political constitution, as the balance of power between the different ideological forces of a political community changes, these factions rotate in and out of power. Consequently, those utopian ideas of preservation and or modernisation can become the dominant ideology, guiding how political constitution is maintained. They will, however, be challenged by new utopian visions held by those who are out of power.

From this perspective, the Labour Party’s post-1997 constitutional reform agenda can be understood as an attempt to preserve and modernise the UK political constitution in response to the changing political, social and economic conditions following the rise of neo-liberalism and perceived constitutional problems during 18 years of Conservative rule. Rather than an ideological abandonment of the political constitution, the conscious introduction of weak-form judicial review under the Human Rights Act coupled with increased political scrutiny of the human rights implication of bills within government and Parliament are better understood as an ideological response to the perceived problems of the then political constitution.^186^ Similar reforms that open up new sites of political contestation, transparency and accountability via the introduction of devolution and the Freedom of Information Act can also be understood as an attempt to rejuvenate the political constitution. The UK’s political constitution was reconfigured to meet the needs of the day. This is evident in the fact that it retains its predominantly political character, even though more legal aspects were blended in.^187^ Similarly, the Conservative Party’s constitutional reform agenda can equally be understood as an attempt to preserve and modernise the political constitution by aligning it with their respective utopian vision developed during their time out of office.^188^

Of course, one can still argue whether these Labour and Conservative governments correctly perceived the challenges facing the UK’s predominantly political constitution at their respective times and whether their attempts to preserve and modernise were necessary and/or effective. My point is simply that both approaches to the constitution can be understood as examples of how different political ideologies influence and guide the behaviour of political actors in the preservation and modernisation of a political constitution in response to changing political, social and economic circumstances. We should recognise a political constitution is sustained and remains dynamic through the continuous struggle between the different ideological readings of political constitutionalist thought, which shape and inform how constitutional actors navigate political, social and economic tensions that participate in the preservation and modernisation of their political constitution.

## 8. Conclusion

Let us return to the question with which this article started: how is ordinary democratic politics understood by political constitutionalists? The answer depends on the political constitutionalist you ask and the political ideology that they adhere to. Various political ideologies have influenced the development of political constitutionalism, leading to different understandings of law, democracy and government. The democratic socialist and conservative readings focus on the issues of change. In contrast, the liberal and republican readings are concerned with the issues of consent and contestability in the social contract. Consequently, political constitutionalists decode constitutional events differently from one another, disagreeing on issues of government power, accountability and constitutional reform.

Whether political constitutionalism is compatible with an ideology depends on whether political constitutionalism is logically and culturally compatible with the ideology. Political constitutionalism may logically be incompatible with some ideologies. Political constitutionalism may appeal to certain ideologies within the UK’s constitutional culture but have little appeal to their counterparts in other constitutional cultures.

Since constitutional theories are often instrumentally used in politics to justify ideological preferences, it is crucial to reflect on whether these claims are compatible with political constitutionalism. Are they central, peripheral or abusive examples of political constitutionalism? This can be challenging because political constitutionalism often appears as a thin constitutional theory. A thin conception is only justifiable when used as an analytical device for characterising constitutions and reflecting on constitutional change. In taking a critical interpretive approach, we can uncover and challenge those who use a thin conception merely to mask their ideological positions, particularly when those claims under closer scrutiny are revealed to be incompatible or even detrimental to political constitutionalism.

In conclusion, political constitutionalism may appeal to a range of ideologies, resulting in different conceptions of ordinary democratic politics. For political constitutionalists, the fact that there is such disagreement about political constitutionalism should be neither surprising nor troubling. It is unsurprising given the pivotal role that reasonable disagreement plays in political constitutionalist thought and is key to sustaining a real-world political constitution.

## Footnotes

1 Graham Gee and Grégoire CN Webber, ‘What Is a Political Constitution?’ (2010) 30 OJLS 273, 287–90.

2 See Mark Tushnet, *Taking the Constitution Away from the Courts* (3rd edn, Princeton UP 2000); Mark Tushnet, ‘Progressive Constitutionalism: What Is It’ (2011) 72 Ohio St LJ 1073.

3 See Peter C Oliver, ‘“A Constitution Similar in Principle to That of the United Kingdom”: The Preamble, Constitutional Principles, and a Sustainable Jurisprudence’ (2019) 65 McGill LJ 207; Michael Pal, ‘The Unwritten Principle of Democracy’ (2019) 65 McGill LJ 296.

4 See Janet McLean, ‘The Unwritten Political Constitution and Its Enemies’ (2016) 14 ICON 119.

5 See Su Bian, ‘Political but Incontestable: A Review of “Political Constitutionalism” in China’ (2021) 10 GlobCon 10.

6 See Jhaveri Swati, Tarunabh Khaitan and Dinesha Samararatne (eds), *Constitutional Resilience in South Asia* (Hart Publishing 2023).

7 For a robust defence of the interpretive approach to public law, see Martin Loughlin, ‘Theory and Values in Public Law: An Interpretation’ [2005] PL 48, 61–2; Martin Loughlin, ‘Constitutional Theory: A 25th Anniversary Essay’ (2005) 25 OJLS 183, 186.

8 On the liberal leanings of Legal Constitutionalism, see Thomas Poole, ‘Back to the Future? Unearthing the Theory of Common Law Constitutionalism’ (2003) 23 OJLS 435.

9 cf JAG Griffith, ‘The Political Constitution’ (1979) 42 MLR 1; Graham Gee, ‘The Political Constitution and the Political Right’ (2019) 30 KLJ 148, 155.

10 This overview is not too dissimilar to Marco Goldoni’s overview of political constitutionalism. See Marco Goldoni, ‘Two Internal Critiques of Political Constitutionalism’ (2012) 10 ICON 926, 928–37.

11 See Jürgen Habermas, ‘Constitutional Democracy: A Paradoxical Union of Contradictory Principles?’ (2001) 29 PT 766.

12 Political constitutionalists are admittedly vague about what the precise role of the courts should be. cf JAG Griffith, ‘The Common Law and the Political Constitution’ (2001) 117 LQR 42; Jeremy Waldron, ‘The Core of the Case Against Judicial Review’ (2006) 115 Yale LJ 1346; Adam Tomkins, ‘The Role of the Courts in the Political Constitution’ (2010) 60 UTLJ 1; Adam Tomkins, ‘What’s Left of the Political Constitution?’ (2013) 14 German Law Journal 2275.

13 The classic distinction between strong-form and weak-form judicial review is that ‘in strong-form judicial review, the courts have general authority to determine what the Constitution means … the mark of weak-form judicial review is that ordinary legislative majorities can displace judicial interpretations of the constitution’. See Mark Tushnet, ‘Alternative Forms of Judicial Review’ (2003) 101 Mich L Rev 2781. Weak-form judicial review is generally accepted as compatible with political constitutionalism, see Waldron, ‘The Core of the Case’ (n 12); Richard Bellamy, ‘Political Constitutionalism and the Human Rights Act’ (2011) 9 ICON 86.

14 cf with Legal/Common Law Constitutionalism. See Poole (n 8); Alexander Latham-Gambi, ‘Political Constitutionalism and Legal Constitutionalism—an Imaginary Opposition?’ (2020) 40 OJLS 737, 742–3.

15 Graham Gee, ‘The Political Constitutionalism of JAG Griffith’ (2008) 28 LS 20, 25; Grégoire CN Webber, *The Negotiable Constitution: On the Limitation of Rights* (CUP 2009) 39.

16 Marco Goldoni and Chris McCorkindale, ‘Three Waves of Political Constitutionalism’ (2019) 30 KLJ 74, 79–82; Goldoni (n 10) 929.

17 See Rosalind Dixon and David Landau, *Abusive Constitutional Borrowing* (OUP 2021) 158–66.

18 Paul Blokker, ‘Populism as a Constitutional Project’ (2019) 17 ICON 535, 547.

19 Gee and Webber (n 1) 286–90.

20 ibid 287.

21 Stephen Tierney, ‘Whose Political Constitution? Citizens and Referendums’ (2013) 14 German Law Journal 2185, 2187.

22 See WB Gallie, ‘Art as an Essentially Contested Concept’ (1956) 6 The Philosophical Quarterly 97.

23 John Rawls, *A Theory of Justice* (Harvard UP 1971) 5–10.

24 Daniel Skeffington, *The Political Constitution: An Idea Worth Protecting?* (The Constitution Society 2022) 13–14.

25 Ben Stanley, ‘The Thin Ideology of Populism’ (2008) 13 Journal of Political Ideology 95, 95.

26 Terry Eagleton, *Ideology an Introduction* (3rd edn, Verso 2007) 1–2.

27 On this trend, see Andrew Halpin, ‘Ideology and Law’ (2006) 11 Journal of Political Ideology 153.

28 Michael Freeden, *Ideologies and Political Theory : A Conceptual Approach* (OUP 1996) 50–1, 54.

29 ibid 77–83.

30 ibid 77–88.

31 ibid 74.

32 ibid 77.

33 ibid.

34 ibid 68–72.

35 Gee, ‘The Political Constitution and the Political Right’ (n 9) 148.

36 Griffith, ‘The Political Constitution’ (n 9); JAG Griffith, ‘The Rights Stuff’ [1993] Socialist Register 106. See also Martin Loughlin, ‘The Political Constitution Revisited’ (2019) 30 KLJ 5.

37 KD Ewing, ‘The Resilience of the Political Constitution’ (2013) 14 German Law Journal 2111.

38 For a critique of this approach, see Ralph Miliband’s attack on democratic socialism’s embrace of the UK’s political model. For Miliband, the UK’s political constitution institutionalised a capitalist democracy, a tool for the preservation of capitalism. By embracing political constitutionalism, democratic socialism and its parliamentary vehicle, the Labour Party had been assimilated and manipulated by capitalist interests to contain conflicts about capitalism. Through sanctioning or supressing left-wing critics and dissents in exchange for the possibility of winning an election, democratic socialists were blunting the workers’ ability to overthrow capitalism. Ralph Miliband, *Capitalist Democracy in Britain* (OUP 1984).

39 Tony Wright, *Socialisms: Old and New* (Routledge 2006) 24–6; Freeden (n 28) 430–3; George Bernard Shaw, *What Socialism Is* (Fabian Society 1890) 13.

40 Richard Henry Tawney, *Equality* (4th edn, George Allen & Unwin 1938) 182.

41 Sidney Webb, *Socialism in England* (Swan Sonnenschein 1908) 9.

42 ibid 9–10.

43 Robert Leach, *British Political Ideologies* (Prentice Hall 1996) 139.

44 Peter Dorey, *The Labour Party and Constitutional Reform: A History of Constitutional Conservatism* (Palgrave Macmillan 2008) 3–4, 349–52; Geoffery Foote, *The Labour Party’s Political Thought: A History* (Palgrave Macmillan 1997) 29.

45 One notable exception is the Webbs’ unusual proposal for a radical overhaul of the British parliamentary system. The Webbs advanced that there should be two parliaments: a ‘social parliament’ and ‘political parliament’. Sidney Webb and Beatrice Webb, *A Constitution for the Socialist Commonwealth of Great Britain* (Longmans, Green and Co 1920). However, the norm was that British socialists advocated minor modifications. See Dorey (n 44); Mark Evans, *Constitution-Making and the Labour Party* (Palgrave Macmillian 2003); Anthony Wright, ‘British Socialists and the British Constitution’ (1990) 43 Parliamentary Affairs 322.

46 Webb (n 41) 118–19.

47 Of course, Eduard Bernstein is not a political constitutionalist, but his claims profoundly influenced many of the British socialists who inspired many modern democratic socialist political constitutionalists. For an account of Bernstein relationship with the Fabians, see Roger Fletcher, ‘Bernstein in Britain: Revisionism and Foreign Affairs’ (1979) 1 The International History Review 349.

48 Eduard Bernstein, *Bernstein: The Preconditions of Socialism* (first published 1899, CUP 1993) 140.

49 ibid 153.

50 ibid 143–4.

51 ibid 142.

52 EFM Durbin, *The Politics of Democratic Socialism: An Essay on Social Policy* (first published 1940, Routledge 2019) 271.

53 ibid; Clement Attlee, *The Labour Party in Perspective* (Victor Gollancz 1937) 117.

54 Aneurin Bevan, *In Place of Fear* (Quartet Books 1978) 29.

55 Attlee (n 53) 113–18.

56 Bevan (n 54) 25.

57 Bernstein (n 48) 166.

58 KD Ewing, *The Cost of Democracy: Party Funding in Modern British Politics* (Hart Publishing 2007) 27–8.

59 Richard Stafford Cripps, ‘Can Socialism Come by Constitutional Methods?’ in C Addison and others, *Problems of a Socialist Government* (Victor Gollancz 1933) 49.

60 Ewing, ‘The Resilience of the Political Constitution’ (n 37) 2118.

61 Dorey (n 44) 15.

62 JAG Griffith, *The Politics of the Judiciary* (4th edn, Fontana Press 1991) 19–20, 255, 319; more recently, see Richard Ekins and Graham Gee (eds), *Judicial Power and the Left* (The Policy Exchange 2017).

63 KD Ewing, ‘The Unbalanced Constitution’ in Tom Campbell, KD Ewing and Adam Tomkins (eds), *Sceptical Essays on Human Rights* (OUP 2001) 105.

64
*Taff Vale Railways Co v Amalgamated Society of Railway Servants* [1901] AC 426.

65
*Amalgamated Society of Railway Servants v Osborne* [1910] AC 87.

66
*Roberts v Hopwood* [1925] AC 578.

67 Robert Francis Vere Heauston, *Lives of the Lord Chancellors 1885–1940* (Clarendon Press 1964) 76. For a broad history, see Griffith, *The Politics of the Judiciary* (n 62) 75–150.

68 Griffith, *The Politics of the Judiciary* (n 62) 328.

69 Dorey (n 44) 14, 26, 358; Danny Nicol, ‘Professor Tomkins’ House of Mavericks’ [2006] PL 467, 470–2.

70 Dorey (n 44) 52, 98, 359.

71 KD Ewing, ‘The Law and the Constitution: Manifesto of the Progressive Party’ (2004) 67 MLR 734, 735.

72 Jeremy Waldron, *Law and Disagreement* (OUP 1999); Jeremy Waldron, *Political Political Theory* (Harvard UP 2016).

73 Aileen Kavanagh, *Constitutional Review under the UK Human Rights Act* (OUP 2009) 371; see also Cormac Mac Amhlaigh, ‘Putting Political Constitutionalism in Its Place’ (2016) 14 ICON 175.

74 In 1996, Waldron was invited to give the John Robert Seeley Lectures, in which Waldron defended the constitutional importance of legislation within legal thought. These lectures were published as *The Dignity of Legislation* in 1999. In the same year, Waldron would also publish his seminal *Law and Disagreement*, which builds upon many of themes found within the *Dignity of Legislation*. See Jeremy Waldron, *The Dignity of Legislation* (CUP 1999); Waldron, *Law and Disagreement* (n 72).

75 Goldoni and McCorkindale (n 16) 79.

76 Jeremy Waldron, ‘Theoretical Foundations of Liberalism’ (1987) 37 The Philosophical Quarterly 127, 135–6.

77 ibid 133.

78 ibid 133–4.

79 Waldron, *Law and Disagreement* (n 72) 102.

80 ibid 232–54.

81 ibid 223.

82 ibid 88–146, 308–12.

83 ibid 309.

84 Waldron, *Political Political Theory* (n 72) 125–144.

85 Waldron, *Law and Disagreement* (n 72) 268.

86 ibid 221–2.

87 ibid 292, 303.

88 ibid 101.

89 ibid 32. For an institutionalist critique of this approach, see Keith E Whittington, ‘In Defense of Legislatures’ (2000) 28 PT 690; Robert Greally, ‘Why Political Institutions (and Institutionalism) Matter’ [2023] PL 81.

90 Waldron, *Political Political Theory* (n 72).

91 ibid 93–124, 149–65.

92 ibid 150, 155–6.

93 ibid 156.

94 ibid 76.

95 See ibid 72–92.

96 ibid 160–1.

97 Richard Bellamy, *Political Constitutionalism: A Republican Defence of the Constitutionality of Democracy* (CUP 2007); Adam Tomkins, *Our Republican Constitution* (OUP 2005).

98 Samantha Besson and José Luis Martí, ‘Law and Republicanism: Mapping the Issues’ in Samantha Besson and José Luis Martí (eds), *Legal Republicanism: National and International Perspectives* (OUP 2009) 3.

99 Phillip Pettit, *Republicanism: A Theory of Freedom and Government* (OUP 1997) 66.

100 ibid 52.

101 ibid 63–4.

102 ibid 55–6.

103 ibid 58.

104 ibid.

105 Cécile Laborde, ‘Republicanism’ in Michael Freeden and Marc Stears (eds), *The Oxford Handbook of Political Ideologies* (OUP 2013) 525.

106 Bellamy, *Political Constitutionalism: A Republican Defence* (n 97) 5, 21–5.

107 ibid 164.

108 ibid 218.

109 ibid 225–6.

110 ibid 230.

111 ibid 191.

112 ibid 234.

113 ibid 9.

114 Tomkins, *Our Republican Constitution* (n 97) 64.

115 ibid 51.

116 ibid 2, 51.

117 ibid 1, 25.

118 Bellamy, *Political Constitutionalism: A Republican Defence* (n 97) 151/ Bellamy does accept a statutory bill of rights, guarded by the courts under weak-form judicial review, see Bellamy, ‘Political Constitutionalism and the Human Rights Act’ (n 13).

119 Bellamy, *Political Constitutionalism: A Republican Defence* (n 97) 5, 165.

120 Tomkins, *Our Republican Constitution* (n 97) 1, 9–10.

121 ibid 1, 25.

122 ibid 27–31.

123 ibid 27–30; Tomkins, ‘The Role of the Courts’ (n 12). For a refinement of his initial arguments, see Tomkins, ‘What’s Left’ (n 12) 2280–9.

124 Tomkins, *Our Republican Constitution* (n 97) 25–7.

125 It should be noted that although Bellamy seems to accept there will be unforeseen issues which legislators must address between elections, he notes in such circumstances, ‘What the electorate can expect, though, is that what governments do is consistent with the overall range of policy aims outlined in the election’. See Bellamy, *Political Constitutionalism: A Republican Defence* (n 97) 239.

126 Tomkins, *Our Republican Constitution* (n 97) 62–3.

127 ibid 136–7.

128 ibid 138. For a democratic socialist critique, see Nicol (n 78).

129 Gee, ‘The Political Constitution and the Political Right’ (n 9); see also Gee, ‘The Political Constitutionalism of JAG Griffith’ (n 15).

130 Richard Ekins, ‘Protecting the Constitution How and Why Parliament Should Limit Judicial Power’ [2019] Policy Exchange 1.

131 Jonathan Sumption, *Trials of the State: Law and the Decline of Politics* (Profile Books 2019). For criticism, see Richard Bellamy, ‘The Limits of Lord Sumption: Limited Legal Constitutionalism and the Political Form of the ECHR’ in NW Barber, Richard Ekins and Paul Yowell (eds), *Lord Sumption and the Limits of the Law* (Hart Publishing 2016); Martin Loughlin, ‘What Would John Griffith Have Made of Jonathan Sumption’s Reith Lectures?’ (2019) 90 The Political Quarterly 785.

132 Loughlin, ‘The Political Constitution Revisited’ (n 36) 18–19; Ewing, ‘The Resilience of the Political Constitution’ (n 37) 2127.

133 Martin Loughlin, *Public Law and Political Theory* (OUP 1992) 140–65, 184–90; David Sugarman, ‘Legal Theory, the Common Law Mind and the Making of the Textbook Tradition’ in William Twining (ed), *Legal Theory and Common Law* (Basil Blackwell 1986) 26–62.

134 Gee, ‘The Political Constitution and the Political Right’ (n 9) 150.

135 ibid 156.

136 ibid 156–7.

137 Freeden (n 28) 341–3; Samuel P Huntington, ‘Conservatism as an Ideology’ (2016) 51 American Political Science Review 454.

138 Freeden (n 28) 332; John Kekes, ‘What Is Conservatism?’ (1997) 72 Philosophy 351, 367.

139 Kekes (n 138) 353, 366; Kieron O’Hara, *Conservatism* (Reaktion Books 2011) 86–90; Gee, ‘The Political Constitution and the Political Right’ (n 9) 156.

140 Anthony Quinton, *The Politics of Imperfection: The Religious and Secular Traditions of Conservative Thought in England from Hooker to Oakeshott* (Faber & Faber 1978) 16; Michael Oakeshott, *Rationalism in Politics and Other Essays* (Methuen & Co 1962) 169.

141 Quinton (n 140) 20; Kekes (n 138) 365–6; O’Hara (n 139) 73, 109.

142 Edmund Burke, *Revolutionary Writings: Reflections on the Revolution in France and the First Letter on a Regicide Peace* (Iain Hampsher-Monk ed, CUP 2014) 19.

143 Kekes (n 138) 367; Gee, ‘The Political Constitution and the Political Right’ (n 9) 159.

144 O’Hara (n 139) 74.

145 Quinton (n 140) 16; O’Hara (n 139) 74.

146 Gee, ‘The Political Constitution and the Political Right’ (n 9) 157; Quinton (n 140) 16.

147 Quinton (n 140) 11, 16–17; Kekes (n 138) 356.

148 Gee, ‘The Political Constitution and the Political Right’ (n 9) 157; O’Hara (n 139) 49–50; see also Graham Gee and Grégoire Webber, ‘Rationalism in Public Law’ (2013) 76 MLR 708.

149 Philip Norton, ‘The Constitution’ in Kevin Hickson (ed), *The Political Thought of the Conservative Party since 1945* (Springer 2005); Ekins (n 130); Gee, ‘The Political Constitution and the Political Right’ (n 9).

150 Norton (n 149) 96–7.

151 Despite Gee suggesting such judgments concerned the conservatives, he provides no evidence to support this claim. Gee (n 9) 167–8. Elsewhere, Gee and Ekins seemingly imply judges did not engage in political decision making prior to the European Communities Act 1972 and the Human Rights Act 1998. See RE Ekins and G Gee, ‘Putting Judicial Power in Its Place’ (2018) 36 UQLJ 375. On the conservative appeal of the common law, see Sugarman (n 133).

152 Loughlin, ‘The Political Constitution Revisited’ (n 36) 18–19.

153 Gee, ‘The Political Constitution and the Political Right’ (n 9) 159–60; Ekins and Gee (n 151) 375–8.

154 Gee, ‘The Political Constitution and the Political Right’ (n 9) 160; Ekins and Gee (n 151) 378. For a critical take on this conservative assessment, see Loughlin, ‘The Political Constitution Revisited’ (n 36) 18–19.

155 See the European Communities Act 1972 and the Human Rights Act 1998.

156 Quinton (n 140) 13, 20.

157 ibid 20; Gee, ‘The Political Constitution and the Political Right’ (n 9) 157–8.

158 Kekes (n 138) 367; Gee, ‘The Political Constitution and the Political Right’ (n 9) 158.

159 See Herbert Döring, ‘Time as a Scarce Resource: Government Control of the Agenda’ in Herbert Döring (ed), *Parliaments and Majority Rule in Western Europe* (St Martin’s Press 1996) 223–46.

160 Gee, ‘The Political Constitution and the Political Right’ (n 9) 149, 157–8; Norton (n 149); Stephen Laws and Richard Ekins, ‘Endangering Constitutional Government: The Risks of the House of Commons Taking Control’ (2019) <https://policyexchange.org.uk/wp-content/uploads/2019/03/Endangering-Constitutional-Government.pdf> accessed 26 May 2021.

161 Gee, ‘The Political Constitution and the Political Right’ (n 9) 160.

162 Edmund Fawcett, *Conservatism: The Fight for a Tradition* (Princeton UP 2020) 48–9, 56–7.

163 Robert Eccleshall, ‘English Conservatism as Ideology’ (1977) 25 Political Studies 62, 80–1.

164 Gee, ‘The Political Constitution and the Political Right’ (n 9) 159–60.

165 This broadly aligns with the Peelite narrative of executive–legislative relations. See Anthony Harold Birch, *Representative and Responsible Government: An Essay on the British Constitution* (Allen & Unwin 1964) 75–8.

166 Goldoni (n 10) 940–3.

167 Joseph A Schumpeter, *Capitalism, Socialism and Democracy* (Taylor & Francis 2010) 242.

168 ibid 244.

169 Leo Amery, *Thoughts on the Constitution* (OUP 1947) 20–1.

170 Gee, ‘The Political Constitution and the Political Right’ (n 9) 161.

171 Norton (n 149) 108.

172 Griffith, ‘The Political Constitution’ (n 9).

173 Gee, ‘The Political Constitution and the Political Right’ (n 9).

174 Griffith, ‘The Political Constitution’ (n 9) 1–2, 19–20.

175 ibid 19–20.

176 ibid 19.

177 See Loughlin, ‘The Political Constitution Revisited’ (n 36).

178 Gee, ‘The Political Constitution and the Political Right’ (n 9) 155.

179 ibid.

180 ibid 155–6.

181 Martin Loughlin, ‘The Constitutional Imagination’ (2015) 78 MLR 1, 11–12.

182 Dieter Grimm, *Constitutionalism: Past, Present, and Future* (OUP 2016) 149. In the UK context, see Nick Barber, ‘Against a Written Constitution’ [2008] PL 11, 16–18.

183 Martin Loughlin, ‘The Silences of Constitutions’ (2018) 16 ICON 922, 924–5.

184 Loughlin, ‘The Constitutional Imagination’ (n 181) 13.

185 Although Loughlin invokes the term ideology in its Marxist sense, there is nothing in his argument which prevents a non-Marxist reading of ideology being used to reach the same conclusion about constitutional imagination. ibid 13–14.

186 Home Office, *Rights Brought Home: The Human Rights Bill* (Cm 3782, 1997) paras 2.10–2.15.

187 Tomkins, ‘What’s Left’ (n 12) 2276, 2289.

188 Ekins (n 130).

